# 

**Published:** 1856-10

**Authors:** 


*To Subscribers.*

The mailing of the September number of the *Journal* was delayed two weeks for the purpose of enclosing bills to subscribers.

There is over $1,800 due us on Volumes IV. and V. of the *Journal.* Two or four dollars is but a small sum, which would hardly be missed by any individual subscriber; but $1,800 is a large item for us to wait for.

### RECEIPTS ON SUBSCRIPTIONS,

FROM AUG. 17TH TO OCT. 5TH, 1856.

### ILLINOIS.

| | | | |
|---|---|---|---|
| Dr. E. A. Rogers, - | Vol. 5, | $2,00 |
| " Norcom, - - | " | 5, | 2,00 |
| " Gore & Amerman, | " | 6, | 2,00 |
| " Hamill, - - - | " | 5, | 2,00 |
| " J. B. Brown, - - | " | 5, | 2,00 |
| Jonesboro Medical Soc. | " | 5, | 2,00 |
| " C. E. Smith, - | " | 5, | 2,00 |
| " J. Welch, - - | " | 5, | 2,00 |
| " E. Smith, - | " | 5, | 2,00 |
| " W. E. Rennolds, - | " | 5, | 4,00 |
| " H. C. Morey, - | " | 5, | 2,00 |

### INDIANA.

| | | | |
|---|---|---|---|
| Dr. Owen Thomas, - | Vol. | 4, | $3,00 |
| " D. Rea, - - | " | 5, | 2,00 |
| " A. Chapman, - | " | 5, | 2,00 |

### WISCONSIN.

| | | | |
|---|---|---|---|
| Dr. L. C. Bicknell, | Vol. | 4, 5, | 4,00 |
| " E. G. Horton, - | " | 5, | 2,00 |

### IOWA.

| | | | |
|---|---|---|---|
| Dr. J. Y. Higbee, - | Vol. | 5, | $2,00 |

### MICHIGAN.

| | | | |
|---|---|---|---|
| Dr. S. W. Lovell, - | Vol. | 5. | $2,00 |

